# Exploring the ability of stroke survivors in using the contralesional hemisphere to control a brain–computer interface

**DOI:** 10.1038/s41598-022-20345-x

**Published:** 2022-09-28

**Authors:** Salem Mansour, Joshua Giles, Kai Keng Ang, Krishnan P. S. Nair, Kok Soon Phua, Mahnaz Arvaneh

**Affiliations:** 1 Department of Automatic Control and Systems Engineering, University of Sheffield, Mapping Street, Sheffield, S13JD UK; 2 Agency for Science Technology and Research, Institute for Infocomm Research, Singapore, Singapore; 3 School of Computer Science and Engineering, Nanyang Technological University, Singapore, Singapore; 4 Neurology, Royal Hallamshire Hospital, Sheffield Teaching Hospitals NHS Foundation Trust and The University of Sheffield, Sheffield, UK

**Keywords:** Stroke, Biomedical engineering

## Abstract

Brain-computer interfaces (BCIs) have recently been shown to be clinically effective as a novel method of stroke rehabilitation. In many BCI-based studies, the activation of the ipsilesional hemisphere was considered a key factor required for motor recovery after stroke. However, emerging evidence suggests that the contralesional hemisphere also plays a role in motor function rehabilitation. The objective of this study is to investigate the effectiveness of the BCI in detecting motor imagery of the affected hand from contralesional hemisphere. We analyzed a large EEG dataset from 136 stroke patients who performed motor imagery of their stroke-impaired hand. BCI features were extracted from channels covering either the ipsilesional, contralesional or bilateral hemisphere, and the offline BCI accuracy was computed using 10 $\times$ 10-fold cross-validations. Our results showed that most stroke patients can operate the BCI using either their contralesional or ipsilesional hemisphere. Those with the ipsilesional BCI accuracy of less than 60% had significantly higher motor impairments than those with the ipsilesional BCI accuracy above 80%. Interestingly, those with the ipsilesional BCI accuracy of less than 60% achieved a significantly higher contralesional BCI accuracy, whereas those with the ipsilesional BCI accuracy more than 80% had significantly poorer contralesional BCI accuracy. This study suggests that contralesional BCI may be a useful approach for those with a high motor impairment who cannot accurately generate signals from ipsilesional hemisphere to effectively operate BCI.

## Introduction

Approximately 65% of stroke survivors experience motor disability lasting beyond six months after stroke^1^. Neuroplasticity which is the brain’s ability to reorganize neuronal connections plays a pivotal role in motor function recovery. Neuroplasticity helps establish other neural pathways to compensate for the brain damage caused by stroke^2–4^. Therefore, it is crucial to develop more effective and efficient rehabilitation approaches that induce neuroplasticity and promote better functional recovery after stroke.

In recent years, brain-computer interfaces (BCI) have attracted considerable attention as a new stroke rehabilitation tool^5,6^. BCI captures, analyzes and interprets brain signals as commands for communication and control. In a BCI-based stroke rehabilitation system, stroke patients perform motor imagery or attempt to move the affected limb (if possible), while the electroencephalogram (EEG) signals are recorded. When motor relevant brain signals are detected during the patient’s imagined/attempted limb movement, an external device will be activated to provide feedback to the patient. The most common type of feedback used in the BCI-based stroke therapy is kinetic feedback, which involves moving the affected hand along a designated path once movement-related EEG are detected. For example, the BCI clinical trials conducted by Ramos et al.^7^ and Biasiucci et al.^8^ used kinetic feedback by moving the affected hand through an exoskeleton and functional electrical stimulation (FES) respectively. Several studies reported that the neurofeedback, as done in the BCI-based rehabilitation systems, may play a key role in inducing neuroplasticity and improving motor functions^9,10^. Interestingly, a recent meta-analysis reported the superior efficacy of the BCI rehabilitation intervention in improving upper-limb functions in both chronic and subacute stroke patients^11^.

In several BCI clinical studies, the provided neurofeedback was based on the activity of the ipsilesional motor cortex^12–14^. These studies are aligned with functional magnetic resonance imaging (fMRI) and transcranial magnetic stimulation (TMS) studies, confirming that enhancing the excitability of the ipsilesional motor cortex can play an important role in motor recovery after stroke^15^. On the other hand, other studies reported that enhancing the excitability of the contralesional side appears to play a significant role in motor recovery for a subset of stroke patients^16–18^. Similarly, Kaiser et al.^19^ reported that during motor imagery of the impaired hand, more impaired patients showed higher event-related desynchronizations (ERDs) (i.e. EEG signature of motor tasks) in the contralesional hemisphere when compared with less impaired patients. Antelis et al.^20^ found similar outcomes in stroke patients when they attempted and executed a hand movement. Interestingly, a very recent study demonstrated that for stroke users encountering BCI deficiency, i.e. those with poor conventional BCI accuracy, neuronal modulation was significantly greater in the contralesional hemisphere compared to the ipsilesional hemisphere^21^.

Hence, we hypothesize that for some stroke patients, EEG signals from the contralesional hemisphere may outperform EEG signals from the ipsilesional hemisphere in terms of BCI performance. Physiologically, as the contralesional hemisphere is usually unaffected by stroke, it may implied that many stroke patients should be able to generate brain signals from the contralesional hemisphere in response to imagined or attempted movement of the affected hand^22^. Furthermore, a previous study used EEG signals from the contralesional hemisphere to successfully control a BCI^23^. However, this study was limited to only 10 stroke patients, and the final results were not compared with the results of a conventional BCI system that uses ipsilesional signals. More research is needed to fully understand the effects of other confounding variables that may affect the cortical activation patterns and BCI performance in stroke patients, including lesion location and size, and time since stroke.

In short, this paper aims to address the following questions:

- Are the stroke patients able to meaningfully operate a BCI-based rehabilitation system using EEG signals from only the contralesional hemisphere?
- Is there a difference in the performance of stroke patients in controlling BCI using EEG from the contralesional hemisphere when compared to using EEG from the ipsilesional hemisphere or even from both hemispheres and how much is this different?
- Are there any relationships between the BCI performance and the patient’s demographic data including Fugl-Meyer assessment score and time since stroke?

In this study, EEG signals of 136 stroke patients performing motor imagery of their impaired hands and their respective BCI features were extracted from channels covering either the ipsilesional, contralesional or both hemispheres using the common spatial patterns (CSP) algorithm^24^, the filter bank common spatial patterns (FBCSP) algorithm^25^, and the band power (BP) feature extraction algorithm^26^. In order to reduce the dimensionality of the features, we only used the most discriminative ones by applying the mutual information-based best individual feature (MIBIF) algorithm for feature selection. Next, the selected features were classified using the naive Bayesian Parzen window (NBPW) classifier^27^. The above mentioned feature extraction and classification algorithms have been commonly used in previous BCI-based stroke rehabilitation clinical trials^8,28,29^. Finally, the average 10-fold cross validation outcomes of the three types of BCI (i.e. ipsilesional, contralesional and bilateral BCI ) were statistically analyzed in terms of BCI accuracy, as well as the impact of the motor impairment and post-stroke time on the BCI performance. We hope that the results of this study will contribute to a deeper understanding of how to promote personalized modulation of neural signals to enhance neuroplasticity, thereby benefiting the stroke patients.

## Results

### ERD/ERS in contralesional and ipsilesional hemisphere

**Figure 1:**
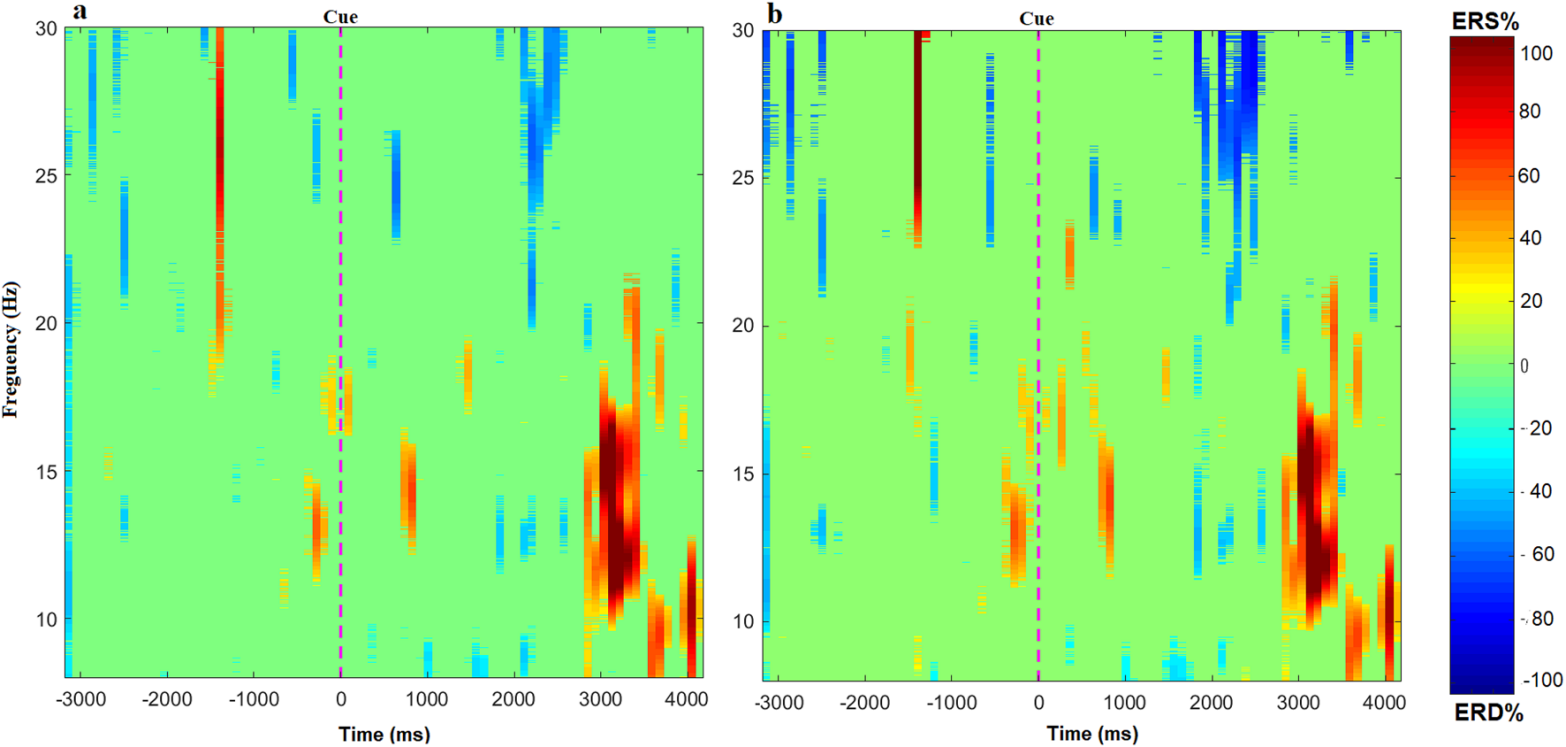
Time-frequency representation shows the grand average of event-related (de)synchronization (ERD/ERS). (**a**) The ERD/ERS in the contralesional hemisphere. (**b**) The ERD/ERS in ipsilesional hemisphere. ERD is indicated by the blue colors, whereas ERS is indicated by the red colors.

**Figure 2:**
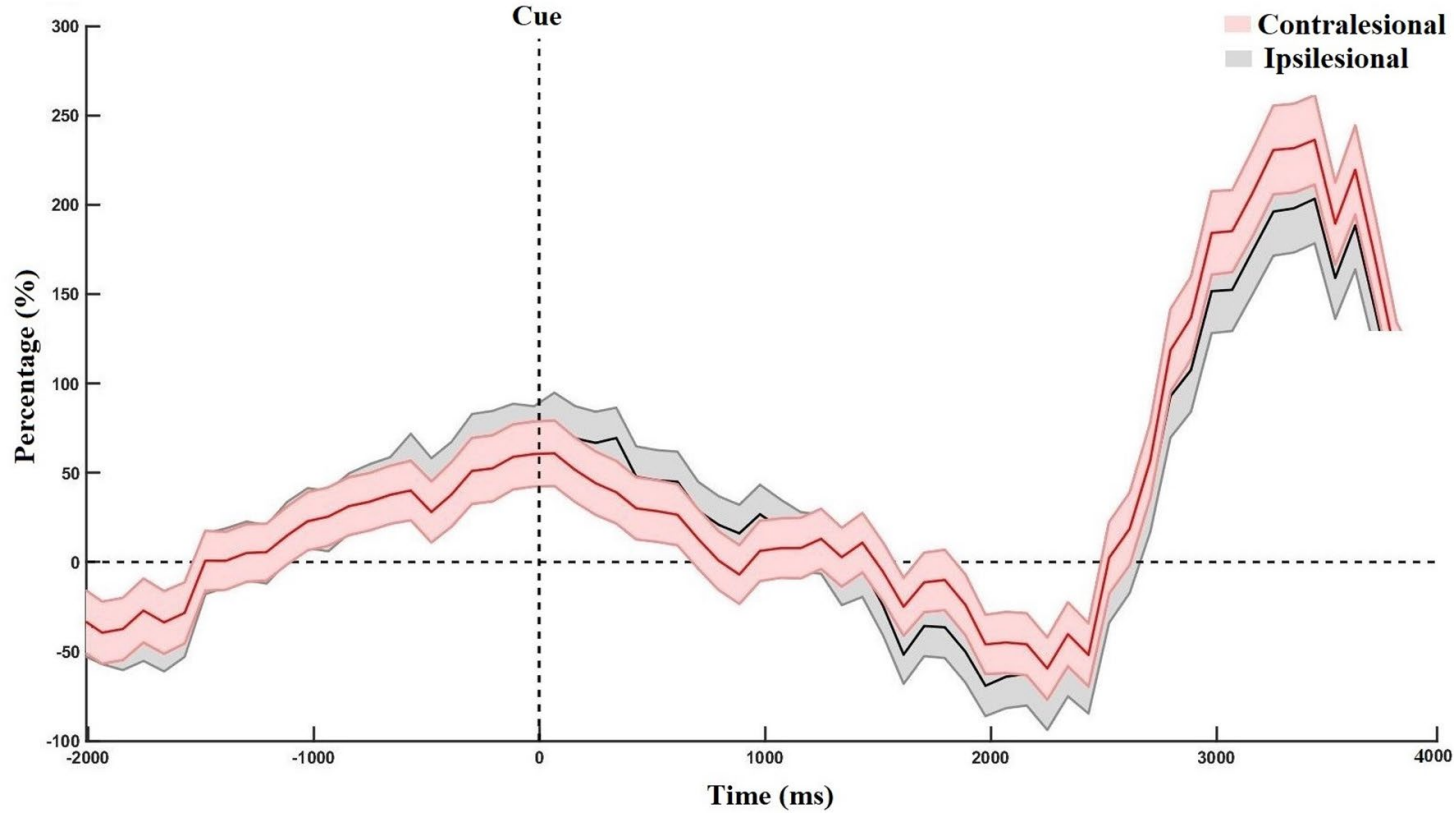
The grand average power change in event-related (de)synchronization (ERD/ERS) in contralesional and ipsilesional hemispheres during motor imagery (i.e. from 0 to 4 s), relative to the resting baseline 1.5 s before the cue.

The time-frequency maps with the ERD/ERS patterns during motor imagery in the contralesional and ipsilesional hemisphere are shown in Fig. 1. We observed that the ERD/ERS phenomenon occurs in both the contralesional and ipsilesional hemispheres. On average, the ipsilesional hemisphere had slightly higher ERD than the contralesional hemisphere, mostly in the beta band. However, the contralesional hemisphere generated a stronger grand average ERS, mostly in the mu-rhythm, compared to ipsilesional hemisphere. Figure 2 shows the grand average power changes in ERD/ERS in the contralesional and ipsilesional hemisphere. It can be observed that during motor imagery there is a relative power decrease (ERD) after onset of motor imagery (t = 0), followed by an increase in the power (ERS) in both hemispheres. The grand average ERD has a slightly lower amplitude in the ipsilesional hemisphere than in the contralesional hemisphere. However, as compared to the ipsilesional hemisphere, the contralesional hemisphere showed a higher amplitude of ERS. Importantly, comparing different time-intervals as well as performing point-to-point comparisons, we did not observe any statistically significant difference between the ERD/ERS of the ipsilesional or contralesional hemispheres over the time range of [0, 4] s (p>0.05,Wilcoxon signed-rank test).

### Comparing classification results of contralesional, ipsilesional and bilateral BCI types

**Table 1:** Comparison of the average $10\times 10$ fold cross-validation BCI accuracies between the three types of BCI (bilateral, contralesional or ipsilesional channels), obtained using three different BCI feature extraction methods.

Feature extraction	Bilateral Acc. $(Mean\pm SD)$	Contralesional Acc. $(Mean\pm SD)$	Ipsilesional Acc. $(Mean\pm SD)$	Bilateral vs Cont. (p-value)	Bilateral vs Ipsi. (p-value)	Cont. vs Ipsi. (p-value)
FBCSP	$74.8\pm 13.02$	$71.23\pm 11.44$	$70.7\pm 12.66$	$< 0.001$	$< 0.001$	0.62
CSP	$69.05\pm 12.59$	$65.87\pm 12.10$	$64.01\pm$ 12.65	$< 0.001$	$< 0.001$	0.029
BP	$74.01\pm 6.9$	$72.52\pm 9.06$	$71.99\pm$9.65	0.18	0.017	0.641

*Acc.* accuracy, *Cont.* contralesional, *Ipsi.* ipsilesional, *SD* standard deviation, *vs* versus.

Supplementary Fig. S1 and Table 1 compare the 10$\times$10-fold cross-validation results of the three types of BCI (i.e. bilateral, contralesional or ipsilesional channels) obtained from 136 stroke patients, using either FBCSP, CSP or BP features. Overall, the use of the bilateral channels with FBCSP features yielded the highest BCI performance, which was significantly better than the ipsilesional and contralesional BCI performance using FBCSP and CSP ($P< 0.001$, Wilcoxon signed-rank test). However, it was not significantly better than the contralesional BCI with BP features ($p > 0.05$, Wilcoxon signed-rank test). The results also showed that, on average, the contralesional BCI performed slightly better than the ipsilesional BCI. Importantly, when using FBCSP and BP feature, there was no statistically significant difference in the stroke patients’ performance in controlling BCI using the ipsilesional hemisphere compared to the one using the contralesional hemisphere.

**Table 2:** Percentage of the patients with the average BCI accuracy (bilateral channels, contralesional, or ipsilesional) less than 60% using different BCI feature extraction methods.

Feature extraction	Contralesional	Ipsilesional	Bilateral
	Below 60%	Below 60%	Below 60%
FBCSP	15.6%	22.79%	13.76%
CSP	28.1%	42.64%	34.1%
BP	7.1%	8.82%	6.6%

Table 2 shows that the overall number of patients who did not achieve an average BCI accuracy above 60% using the contralesional hemisphere was less than the number of patients who failed to achieve an average BCI accuracy above of 60% using the ipsilesional BCI.

**Figure 3:**
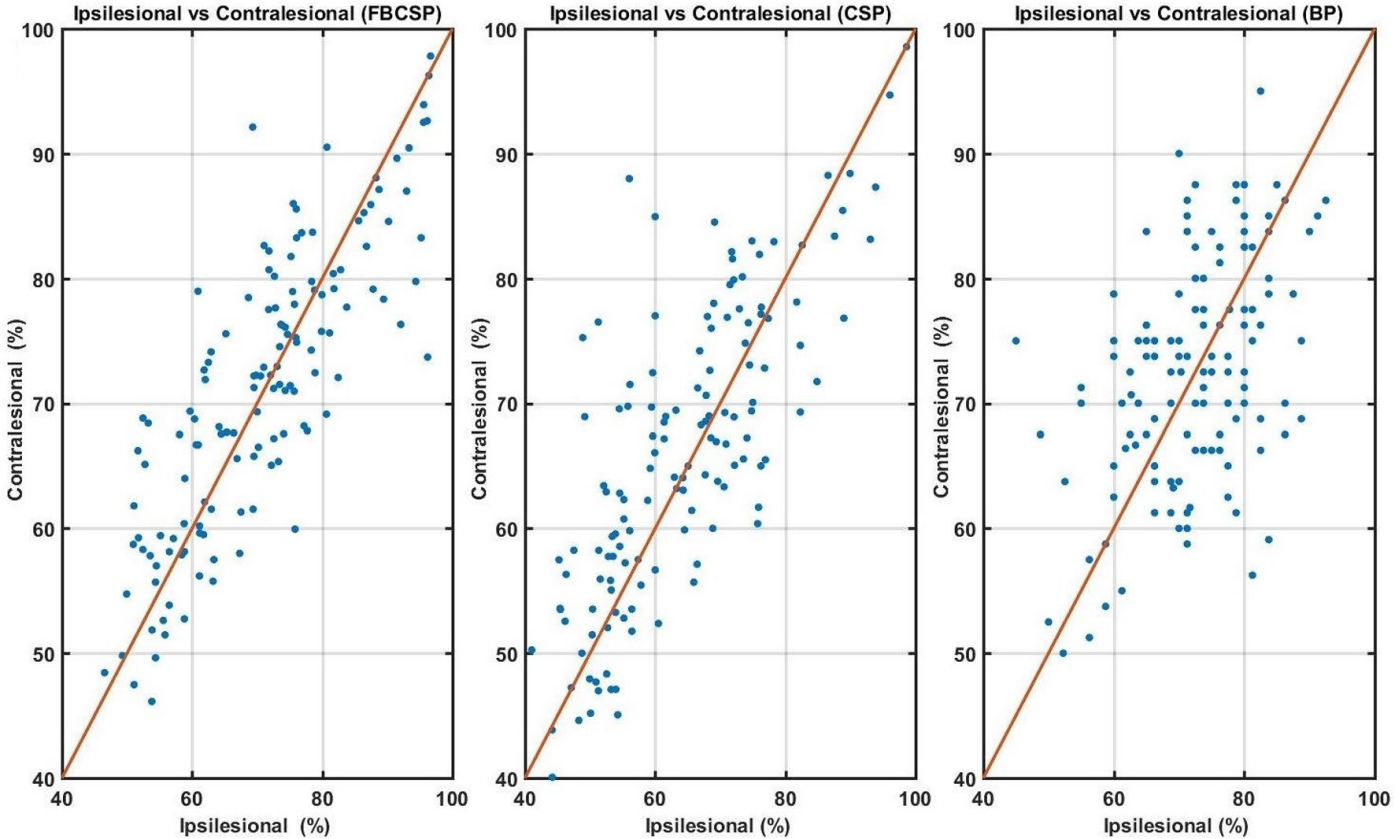
Scatter plots comparing the average cross validation accuracy of contralesional and ipsilesional BCIs using different feature extraction algorithms (FBCSP, CSP, and BP). The blue dots represent the average BCI accuracy for each stroke patient.

**Table 3:** Comparison of the average $10\times 10$ fold cross-validation accuracy of the ipsilesional and contralesional BCI for those with ipsilesional BCI accuracy below 60% and those with the ipsilesional BCI accuracy above 80%, obtained using three different BCI feature extraction methods.

Feature extraction	Ipsilesional Acc.<60%			Ipsilesional Acc.>80%		
	Ipsilesional $(Mean\pm SD)$	Contralesional $(Mean\pm SD)$	p-value	Ipsilesional $(Mean\pm SD)$	Contralesional $(Mean\pm SD)$	p-value
FBCSP	$54.37\pm 3.29$	$57.74\pm 6.51$	0.02	$89.01\pm 5.55$	$83.92\pm 7.33$	$3.8\times 10 ^-5$
CSP	$52.32\pm 4.65$	$57.34\pm 9.57$	$5.05\times 10^-5$	$88.52\pm$ 5.65	$82.62\pm$ 8.55	0.002
BP	$52.27\pm 5.91$	$61.35\pm 8.55$	0.05	$85.05\pm$3.36	$77.08\pm$9.58	$7.79\times 10^-4$

*Acc.* accuracy, *SD* standard deviation.

Interestingly, when we look at the scatter plots in Fig. 3, it can be observed that the contralesional BCI yielded a better classification accuracy than the ipsilesional BCI for those with the the ipsilesional BCI accuracy less than 60% ($p\le 0.05$ for all three feature extraction methods obtained using Wilcoxon signed-rank test). On the contrary, those with the ipsilesional BCI accuracy greater than 80% achieved lower accuracy using the contralesional BCI (p < 0.05, obtained using Wilcoxon signed-rank test). Table 3 provides more details on the corresponding statistical results.

### Impact of post-stroke sensorimotor impairments and the time since stroke on BCI performance

**Table 4:** Comparison of the Fugl-Meyer scores between those with the ipsilesional BCI accuracy below 60% and those with the ipsilesional BCI accuracy over 80%, obtained using three different BCI feature extraction methods.

Feature extraction	Ipsilesional Acc.<60%	Ipsilesional Acc. >80%	
	FMA score	FMA score	p-value
FBCSP	$16.28\pm 16.16$	$29.57\pm 13.84$	0.016
CSP	$18.68\pm 14.24$	$32.54\pm 15.88$	0.015
BP	$19.77\pm 10.96$	$30.33\pm 13.69$	0.047

*FMA* Fugl–Meyer assessment, *Acc.* accuracy.

We did not observe a significant correlation between the ability of stroke patients to use contralesional, ipsilesional or bilateral hemispheres to operate BCI and their Fugl-Meyer score (supplementary Table S1). That being said, we observed significant difference between the Fugl-Meyer scores of those with average ipsilesional BCI accuracy less than 60% and the Fugl-Meyer scores of those with ipsilesional BCI accuracy higher than 80%. From Table 4, we observe that those with the ipsilesional BCI accuracy below 60% had significantly higher motor impairments, measured using Fugl-Meyer assessment, than those with the ipsilesional BCI accuracy above 80% ($p < 0.05$).

Regarding the impact of stroke duration on BCI Performance, we did not observe any significant correlation between the accuracy of detecting motor imagery using either, contralesional, ipsilesional, or bilateral hemisphere and the time since stroke (see supplementary Table S2). Furthermore, no significant difference was found in the time following stroke of patients with the ipsilesional BCI accuracy below 60%, and those with the ipsilesional BCI accuracy above 80% ($p>0.05$).

## Discussion

Many studies showed that in a healthy human, movement of the hand leads to an increased activation in the contralateral motor cortex and a decrease in activation of the ipsilateral motor cortex when compared to the resting state^30^. Although the capacity of modulating ipsilesional brain activity reduces where the damage on the ipsilesional hemisphere is more severe^31^, several BCI clinical studies have shown that many stroke patients are still able to control BCI using EEG signals recorded over the ipsilesional hemisphere^12,13^. Furthermore, a functional imaging study indicated that the ipsilesional hemisphere participated during the motor tasks^32^. This might be because surviving neurons in the ipsilesional cortex are activated during motor tasks^33^.

Interestingly, after stroke undamaged parts of the brain play an adaptive compensatory role, such that movement of the stroke-affected hand may cause an increase in activation of the contralesional motor cortex^34^. Motor attempts and motor imagery are commonly used for stroke recovery using BCI. Brian activation vary among different motor tasks. Tasks involving motor imagery, increased motor impairment was reported to be associated with stronger ERD in the contralesional hemisphere^35^. However, the tasks involving motor attempt ,were associated with higher hemispheric asymmetry in ERS^35^. Nevertheless, people who make good recovery in hand function after a stroke often show relatively normal task-related brain activation in both hemispheres when performing these motor tasks^36^.

It is important to mention that motor execution and motor imagery are complex tasks, involving changes in activity of different parts of the brain including prefrontal, sensory and motor cortex^37^. Prefrontal cortex plays an important role in preparation and planning of movement^38^. Similarly, it is shown that the parietal cortex is involved in high-level cognitive aspects of action control^39^. Stroke often induces widespread brain functional changes and connectivity alterations^40^ (Supplementary Fig. S2 presents examples of the inter-subject variability in brain activation during motor imagery for sex stroke patients). Recent studies observed that motor function recovery in stroke involves not only the corticospinal system but also prefrontal and precortex^37,38^. Thus, the most desirable BCI system for rehabilitation may require the use of a combination of brain signals from the frontal, central and parietal cortex as the BCI control signal.

This study investigated the ability of stroke patients to control the BCI using EEG activity of the contralesional hemisphere. Our results suggest that ERD/ERS phenomenon does occur in both the contralesional and ipsilesional hemispheres. This is further confirming the findings of Antelis et al.^20^, which suggest that the contralesional hemisphere is also involved during the motor imagery of the affected hand.

In addition, the present study finds that the majority of stroke patients are able to operate the BCI using either their contralesional or ipsilesional hemisphere. By comparing the BCI accuracy obtained from the contralesional and ipsilesional hemisphere, we found that patients with the ipsilesional BCI accuracy less than 60% had significantly more motor impairment compared to those with the ipsilesional BCI accuracy greater than 80%. Interestingly, those who achieved the ipsilesional BCI accuracy below 60% achieved a significantly higher contralesional BCI accuracy. Conversely, those who achieved the ipsilesional BCI accuracy greater than 80% had a significantly lower contralesional BCI accuracy. These findings are consistent with previous studies, which indicated that more impaired patients had stronger neural modulations in the contralesional hemisphere than less impaired patients during motor imagery of the affected hand^19–21^.

Conclusively, our study seems to suggest that the use of ipsilesional BCI may lead to a lower BCI accuracy in those patients with severe impairment which offers the use of contralesional BCI as a viable alternative. That being said, future works may include randomized control clinical studies comparing the effects of contralesional and ipsilesional BCI on improving motor function after stroke.

## Materials and methods

### Datasets description

#### Participants

We analyzed the EEG datasets recorded from 136 stroke patients during the BCI screening sessions of four clinical trials^29,41–43^. Among the 136 participants, 17 were in subacute phase (3.32 ± 1.5 months from stroke onset) and 119 in chronic phase (23.68 ± 17.72 months from stroke onset). Participants were 52.81 ± 11.36 years old, on average Fugl-Meyer score was 28.64 ± 12.92.

These four clinical trials were carried out from 1 January 2011, to 30 September 2017 with ethics approval from the institution’s Domain Specific Review Board, National Healthcare Group, Singapore. All four clinical trials are registered on ClinicalTrials.gov as: NCT00955838^29^, NCT01897025^41^, NCT01287975^42^, and NCT02765334^43^. The clinical trial in^29^ investigated the efficacy of the BCI system coupled with the MIT-Manus robotic feedback on upper-limb motor function improvement, whereas the clinical trial in^41^ studied the possible benefits of using transcranial direct current stimulation (tDCS) in combination with the BCI and robotic feedback to improve the motor function. The purpose of the clinical trial described in^42^ was to observe whether the BCI combined with the haptic knob robot can enhance the arm rehabilitation of stroke patients, whereas the effectiveness of BCI with visual feedback for upper-limb stroke recovery as well as the impacts of mental fatigue on BCI performance were investigated in^43^.

Participants were assessed for eligibility based on the following inclusion criteria:

(1) Participants had their first cortical and sub-cortical stroke, with a Fugl-Meyer score ranging from mild to severe impairment of upper extremity function.

(2) Participants could understand the verbal instructions, and achieved a score higher than 6 out of 10 in the abbreviated mental test.

(3) Participants did not suffer from any medical instability, epilepsy, severe depression, skin problems that could get worse due to wearing the EEG cap, severe spasticity in any of the elbow, finger, shoulder or wrist as assessed by the modified Ashworth scale (score $>2$ ), or severe vision problems.

#### Motor imagery-based BCI paradigm

**Figure 4:**
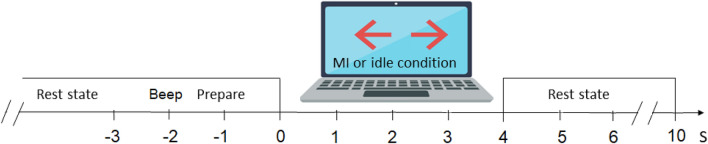
The timing of one trial in the BCI screening session, instructing the patient to perform either motor imagery of stroke-affected hand or idle task.

All participants first attended a motor imagery based BCI screening session without feedback. During the screening session, the participants were instructed to perform motor imagery of their affected arm and hand. The BCI screening session consists of 4 runs and each run consists of 20 trials of the motor imagery task and 20 trials of the idle state in random order. After each run, a 2 min break was given to the participant. On average, each trial took 12 s and each run took about 8 min. Figure 4 illustrates the timing of one trial. A total of 160 trials were collected in each session. The BCI screening session lasted about an hour, including the EEG cap setting.

#### EEG signal acquisition

For the first three clinical trials^29,41,42^, the Neuroscan Nuamps EEG amplifier with unipolar Ag/AgCl electrode channels was used to collect EEG data from 27 channels, which were referenced to the nasion. The collected EEG data was digitally sampled at the frequency of 250 Hz with the resolution of 22 bits and the voltage range of ±130 mV. For the fourth clinical trial^43^, EEG data was collected using the Neurostyle EEG amplifier with 24 unipolar Ag/AgCl electrode channels referenced to the FPz. The EEG was digitally sampled at 256 Hz with a resolution of 24 bits for voltage ranges of ±300mV.

### BCI classification models

**Figure 5:**
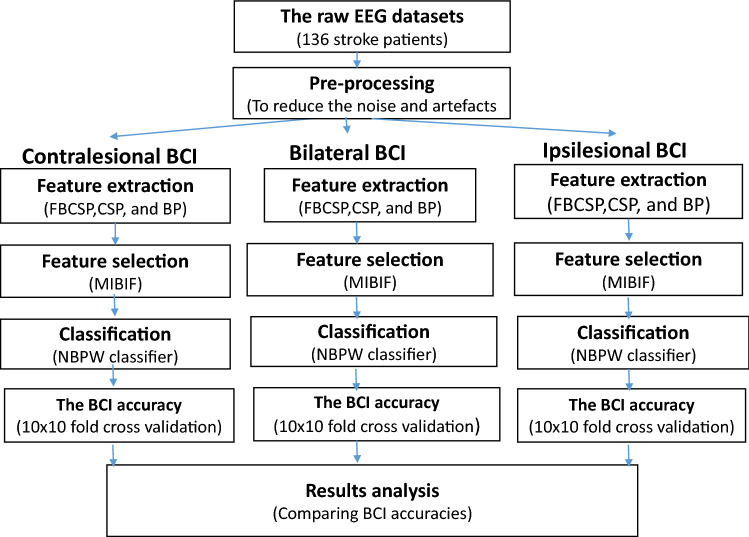
Flowchart presenting the steps taken for training and evaluating the BCI models. *BP* band power, *CSP* common spatial patterns, *FBCSP* filter bank common spatial patterns, *MIBIF* mutual information-based best individual feature selection, *NBPW* Naive Bayesian Parzen window.

Figure 5 shows all the procedures required to training and evaluating the BCI models.

#### Preprocessing and BCI feature extraction

In order to calculate the features, we selected a specific channel set for each type of BCI model (i.e. contralesional, ipsilesional or bilateral BCI):

- Channels that cover either the left or right hemisphere: (FC3, FCz, T7, C3, Cz, CP3, CPz, P3, Pz; or FCz, FC4, Cz, C4, T8, CPz, CP4, Pz, P4 ). Depending on the location of the lesion, they would be called either ipsilesional or contralesional channels.
- Bilateral channels: (FC3, FCz, T7, C3, Cz, CP3, CPz, P3, Pz; FC4, C4, T8, CP4, , P4 ).

In this study, we used the three most commonly used feature extraction algorithms in the BCI-based stroke rehabilitation, namely common spatial patterns (CSP), filter bank common spatial patterns (FBCSP), and band power features (BP)^14,28,29^. When using CSP and BP, we first employed a zero-phase band-pass filter from 8 to 30 Hz in order to clean the raw EEG signal from high-frequency noise and low-frequency artifacts. This frequency band has been selected because it contains the mu (8–12 Hz) and beta (13–30 Hz) rhythms, which are well associated with motor imagery and actual movement^44^. For the FBCSP, we employed a filter bank with nine band-pass filters to partition the EEG dataset into nine frequency bands (4–8 Hz, 8–12 Hz, $\ldots$, and 36–40 Hz)^25^. Four seconds motor imagery and idle class EEG data were extracted after the visual cue for CSP, FBCSP and BP feature extraction. We also extracted 1.5 s of EEG during the preparation period, before the visual cue, as the baseline reference for the BP feature extraction. More detailed information about these feature extraction methods can be found in the subsequent sections. This study was performed without any artifact rejection.

**Common spatial patterns (CSP)** The CSP algorithm has been commonly used in classification of multi-channel EEG signals, recorded during motor imagery^24^. The main concept of CSP is to weight the EEG channels, such that the variance of band-pass filtered EEG signals is maximized in one class and minimized in the other^24^. In this study, the first 2 rows and the last two rows of the CSP matrix were used for spatially filtering the EEG signals. After that, the normalized log variance of the spatially filtered EEG signals were used as the input features for the classifier. Hence, 4 CSP features were extracted in total.

**Filter bank common spatial patterns (FBCSP)** The CSP method can successfully design the optimal spatial filters for distinguishing the two classes of EEG signals in motor imagery-based BCI^45^. However, the efficacy of this method is dependent on its operating frequency band due to the large variability between users^25^. The FBCSP algorithm has been introduced to solve this problem by using a filter bank to filter the EEG data into 9 frequency bands ( i.e. 4–8 Hz, 8–12 Hz, 12–16 Hz, $\ldots$ and 36–40 Hz)^25^. Next, for each frequency band, the band-specific CSP filters are calculated and applied to the corresponding band-passed EEG signals. In this study, for each band, 4 CSP features were extracted using the first and the last two CSP filters. Thus, a total of 36 FBCSP features were extracted.

**Band power features (BP)** The motor imagery and intention of movement can change ongoing brain waves in a form called event-related desynchronization/synchronization (ERD/ERS)^6^. ERD/ERS is characterized by suppression and enhancement of the power of sensorimotor rhythms, respectively, in the frequency range [8–30 Hz]^46^.

In the present study, the BP features measure the average ERD/ERS changes relative to the baseline, as suggested by^47^. After band-pass filtering the EEG signals from 8 to 30 Hz, the BP feature of the $i^{th}$ channel from the $j^{th}$ trial, $\mathbf {BP}(i,j)$, was calculated as

1 $$\begin{aligned} \mathbf {BP}(i,j)= \mathbf {log}\left( \frac{{\mathbf {T}}(i,j)-{\mathbf {B}}(i,j)}{{\mathbf {B}}(i,j)} \times 100\right) , \end{aligned}$$

where ${\mathbf {T}}(i,j)$ denotes the average power of the channel *i* at the trial *j* when performing the task (i.e. 4 s EEG signals immediately after the cue). Similarly, ${\mathbf {B}}(i,j)$ denotes the average power of the channel *i* at the trial *j* during the preparation period (i.e. 1.5 s before the cue).

#### Feature selection

In order to select a more discriminative feature subset from the extracted features, we employed the mutual information-based best individual feature (MIBIF) algorithm based on the filtering feature selection approach^27^. MIBIF calculates the mutual information between each feature and the corresponding class labels, and arranges them in ascending order. Next, the top 4 features with the highest mutual information are selected. In the case of CSP, feature selection was not used because there were only 4 CSP features extracted. Further information about MIBIF can be found in^48^.

#### Classification and validation

In this step, we choose the naive Bayesian Parzen window (NBPW) classifier which is a relatively fast classification algorithm^25,27,49^. The classifier outcomes were objectively evaluated using 10 runs $\times$ 10-fold cross-validation. For each patient, each run of 160 trials is randomly divided into 10 portions. We used nine portions for training and one for testing. This process was repeated ten times, each time saving a different portion for testing. The BCI accuracy was then computed by averaging the 10x10-fold cross validation outcomes.

### Visualization of cortical activity during motor imagery

Event-related synchronization/desynchronization (ERS/ERD) was used to visualize the cortical activation during motor imagery. The grand average time-frequency maps and the grand average ERD/ERS plots were calculated for the ipsilesional and the contralesional hemisphere separately, at either C3 or C4, by pooling the motor imagery trials of all patients. Time-frequency maps are commonly used to visualize the changes in the spectral power of different frequency bands in response to a stimulus across the time^50^. The time-frequency maps were plotted by calculating the power spectrum within a sliding time window and then averaging results across trials. The baseline period for time-frequency maps is 1.5 s before the cue.

To obtain the ERD/ERS plots, the relative change in the relative power with respect to the average power of the preparation period was calculated from 8 to 30 Hz, as presented in^26^. The grand average ERD/ERS plots were presented in time intervals from − 2 to 4 s relative to the onset of the cue, with baseline of 1.5 s before the cue (i.e. preparation period).

### Statistical analysis

We analyzed the data using IBM SPSS Statistics for Windows, released in 2019, version 26.0. In this study, the classification accuracy of the BCI types were compared across the three feature extraction methods using the Wilcoxon rank test. Since our classification accuracy comes from 4 different datasets, we used this non-parametric test^51^.

The 99% confidence of the chance performance for 160 trials is around 60% when using the inverse binomial distribution function^52^. Hence, any participant who has a BCI accuracy of less than 60% is considered to be performing at a chance level. We selected 80% as the other threshold, because in several BCI studies participants with above 80% BCI accuracy were considered as BCI high performers^53^.

We also calculated the correlation between the classification results and the Fugl-Meyer scores as well as the time since stroke for each feature extraction method and each BCI type. Correlation analysis was conducted with Kendall’s Tau correlation, which is a non-parametric method^54^. The significant level was set to *p* = 0.05 for all the analyses.

### Ethical statements

The four clinical trials were approved by the institution’s Domain Specific Review Board, National Healthcare Group, Singapore, and carried out in accordance with the Declaration of Helsinki. Informed written consent was obtained from all participants.

## Supplementary Information

Supplementary Information.

## Data Availability

The data that support the findings of this study are available from Institute for Infocomm Research but restrictions apply to the availability of these data, which were used under license for the current study, and so are not publicly available. Data are however available from the authors upon a reasonable request and with permission of Institute for Infocomm Research and its Institutional review board.
