# Supplementary material for: Exploring the ability of stroke survivors in using the contralesional hemisphere to control a brain–computer interface
Source: Sci Rep. 2022 Sep 28;12:16223. doi: 10.1038/s41598-022-20345-x (PMC9519575; doi:10.1038/s41598-022-20345-x)
Supplement: Supplementary file 1 — Supplementary Information. [file 41598_2022_20345_MOESM1_ESM.docx]

**Exploring the Ability of Stroke Survivors in Using the Contralesional Hemisphere to Control a Brain-computer Interface**

Salem Mansour,, Joshua Giles, Kai Keng Ang, Krishnan P.S. Nair, Kok Soon Phu, and Mahnaz Arvaneh

| 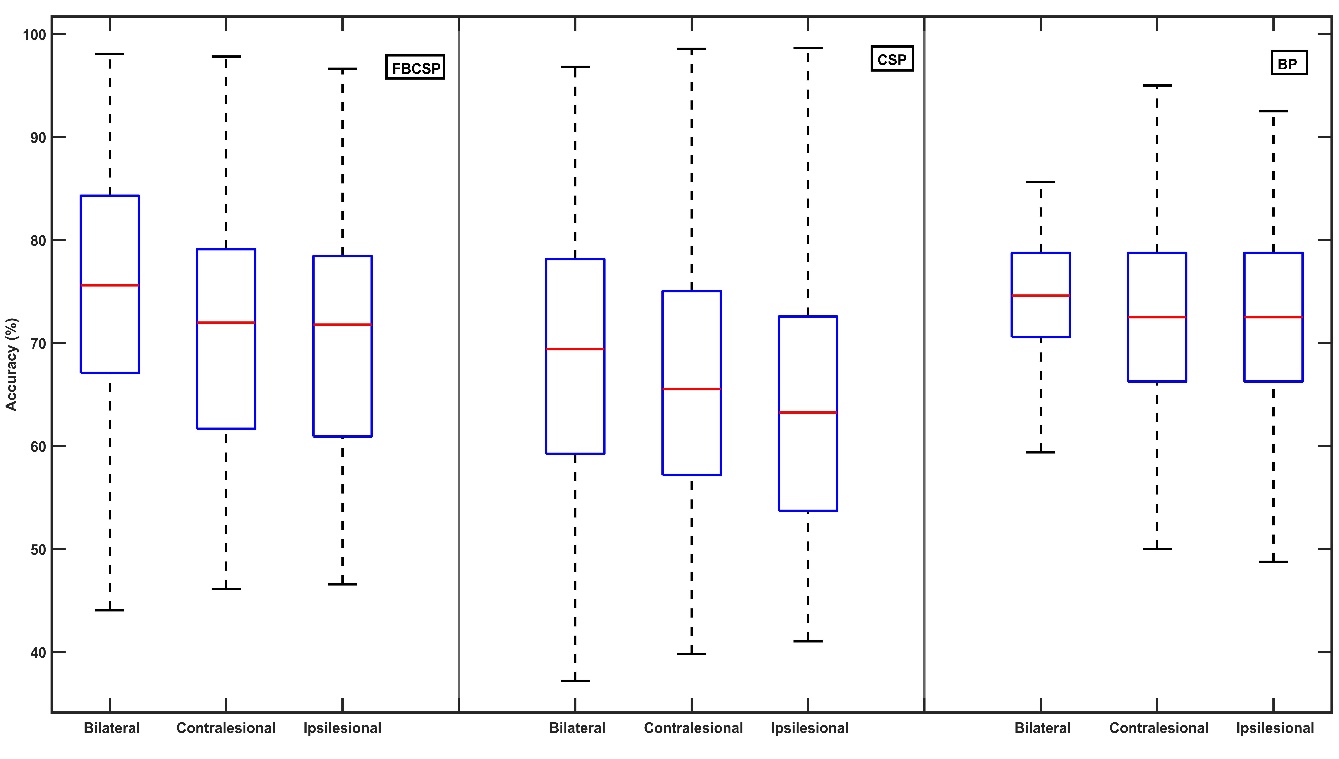 |
| --- |
| Fig. 1. The box-plot shows average cross-validation BCI accuracy of 136 stroke patients using either bilateral channels that cover both hemisphere, contralesional or ipsilesional channels. The y-axis represents the BCI accuracy resulted from 10×10-fold cross-validations, and the x-axis represents the three types of BCI accuracies using either FBCSP, CSP or BP as feature extraction. |

The figure below presents examples of the inter-subject variability in brain activation during motor imagery for 6 stroke patients, obtained using the relevant CSP filter.

As can be seen, participant P01 exhibited significant contralesional sensorimotor brain activation, whereas P06 exhibited ipsilesional sensorimotor activation. Interestingly, P05 presented activation in both ipsilesional and contralesional motor cortex. Results for participants P02, P03, P04, and P06 showed changes in frontal, sensorimotor, and parietal brain activation in the ipsilesional hemisphere. This observation may imply that brain activation is present not only in sensorimotor regions (C3, C4, CP1, CP2, and CP6), but also in parietal regions (P3, P4) and frontal regions (FC6) during the motor imagery of the stroke-affected hand. The BCI based stroke rehabilitation could be able to identify and use this activity to trigger the feedback.

| *P01, The right hand is affected with lesion in the left hemisphere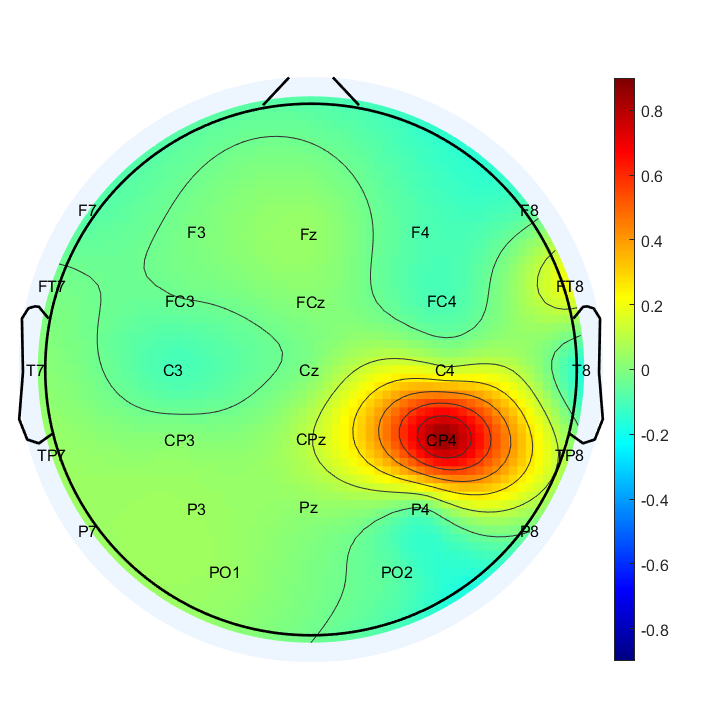* | *P02, The left hand is affected with lesion in the right hemisphere 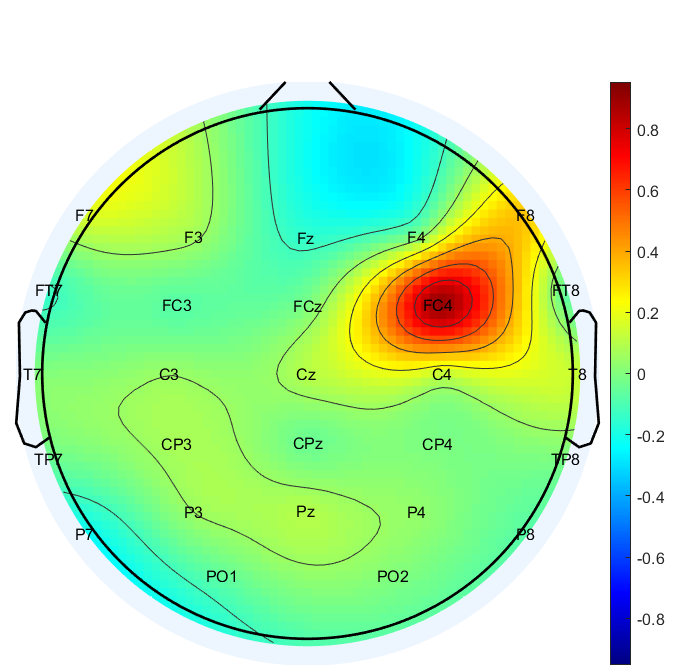* |
| --- | --- |
| *P03, The right hand is affected with lesion in the left hemisphere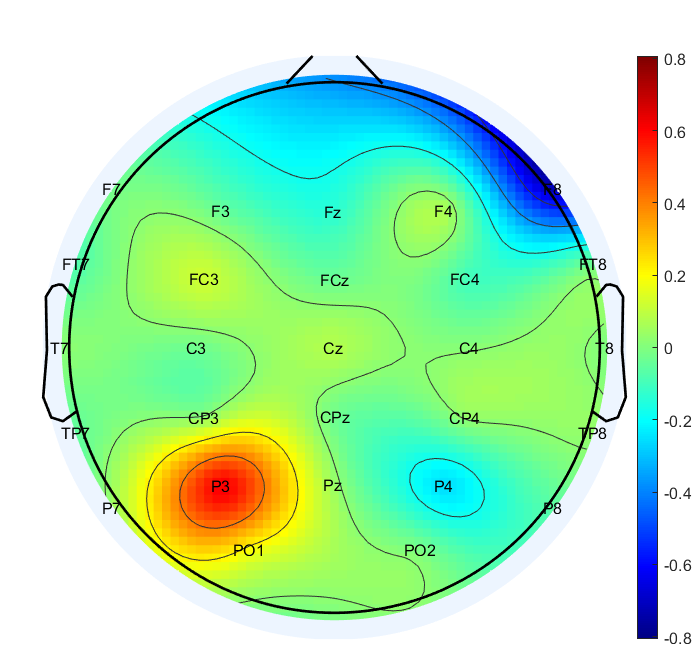* | *P04, The left hand is affected with lesion in the right hemisphere .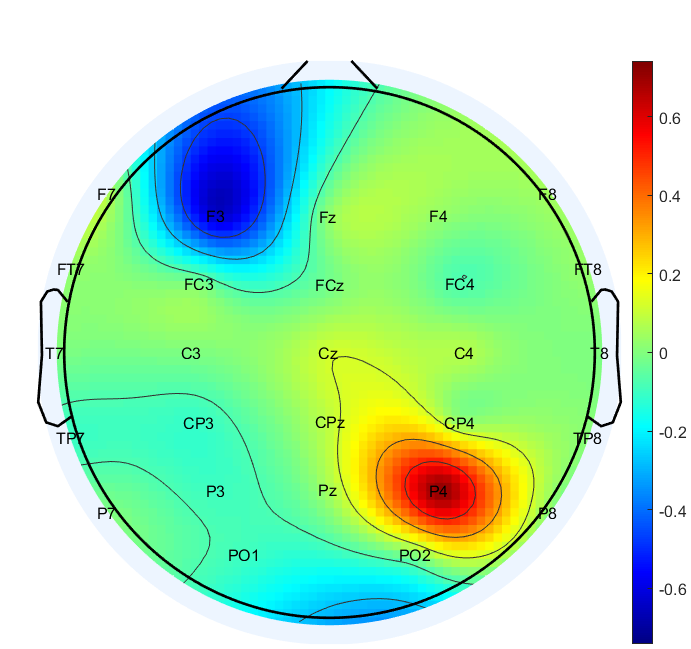* |
| *P05,The right hand is affected with lesion in the left hemisphere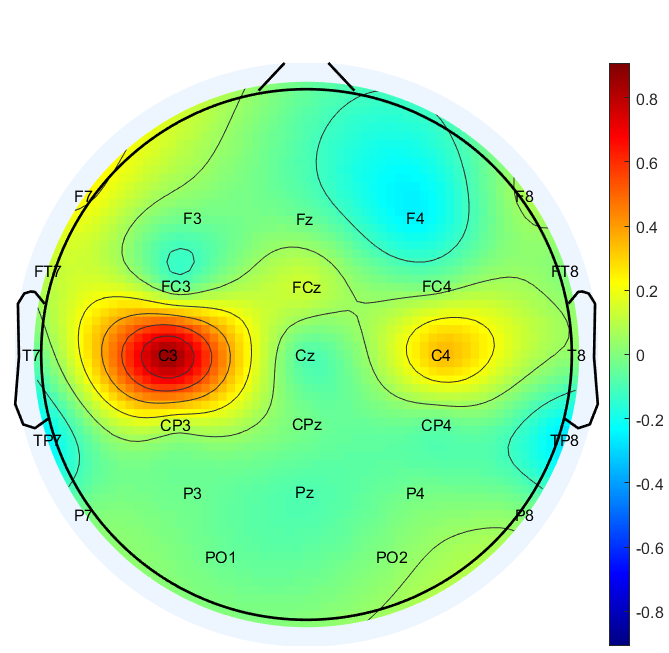* | *P06, The left hand is affected with lesion in the right hemisphere 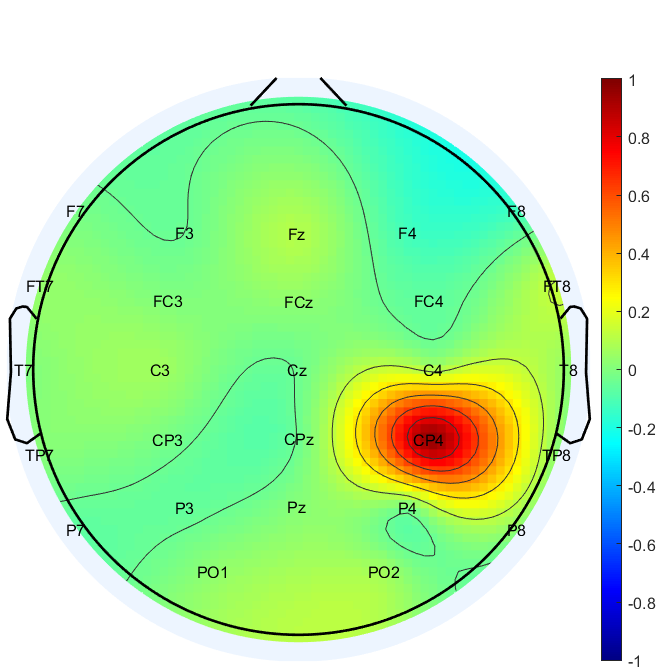* |
| Fig. 2. Scalp maps of six participants showing weights of spatial patterns during motor imagery, obtained using the CSP approach. | |

| Table 1. Correlation between the Fugl-Meyer scores of the patients and their BCI accuracies obtained using either contralesional, ipsilesional, or bilateral channels using three different BCI feature extraction methods. |
| --- |
| 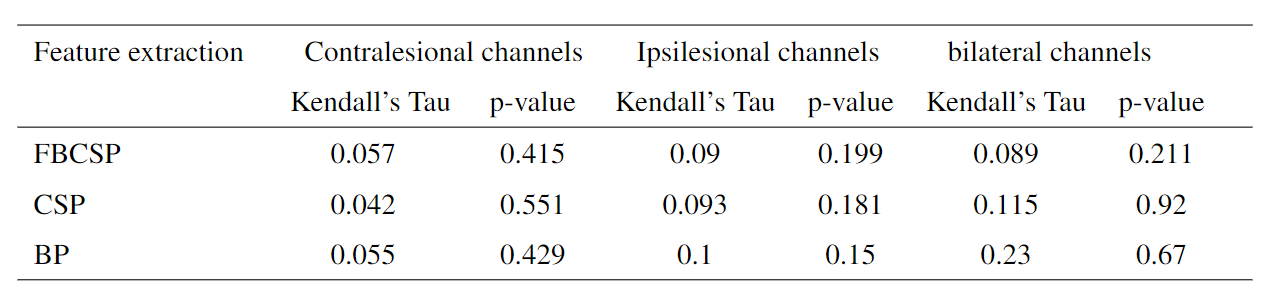 |

| Table 2. Correlation between the time since stroke and the obtained BCI accuracy using contralesional, ipsilesional, or bilateral channels with three different BCI feature extraction methods. |
| --- |
| 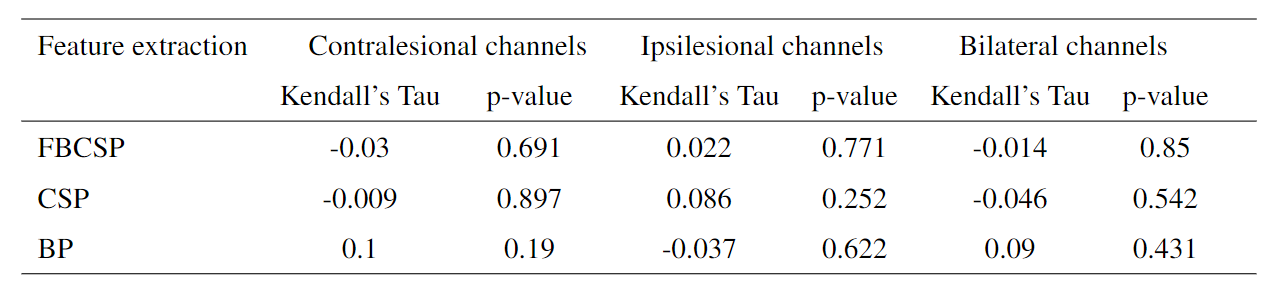 |
